# Altered brain expression and cerebrospinal fluid levels of TIMP4 in cerebral amyloid angiopathy

**DOI:** 10.1186/s40478-024-01823-x

**Published:** 2024-06-24

**Authors:** Lieke Jäkel, Anna M. De Kort, Arno Stellingwerf, Carla Hernández Utrilla, Iris Kersten, Marc Vervuurt, Yannick Vermeiren, Benno Küsters, Floris H. B. M. Schreuder, Catharina J. M. Klijn, H. Bea Kuiperij, Marcel M. Verbeek

**Affiliations:** 1 Department of Neurology, Donders Institute for Brain, Cognition and Behaviour, Radboud University Medical Center, 830 TML, P. O. Box 9101, 6500 HB Nijmegen, The Netherlands; 2 Department of Human Genetics, Radboud University Medical Center, Nijmegen, The Netherlands; 3 Division of Human Nutrition and Health, Chair Group Nutritional Biology, Wageningen University and Research (WUR), Wageningen, The Netherlands; 4 Faculty of Medicine and Health Sciences, Translational Neurosciences, Institute Born-Bunge, University of Antwerp, Antwerp, Belgium; 5 Department of Pathology, Radboud University Medical Center, Nijmegen, The Netherlands; 6 Radboud Alzheimer Center, Radboud University Medical Center, Nijmegen, The Netherlands

**Keywords:** Biomarker, Cerebral amyloid angiopathy, Intracerebral hemorrhage, Tissue inhibitor of metalloproteinases 4

## Abstract

Cerebral amyloid angiopathy (CAA) is a highly prevalent and progressive pathology, involving amyloid-β (Aβ) deposition in the cerebral blood vessel walls. CAA is associated with an increased risk for intracerebral hemorrhages (ICH). Insight into the molecular mechanisms associated with CAA pathology is urgently needed, to develop additional diagnostic tools to allow for reliable and early diagnosis of CAA and to obtain novel leads for the development of targeted therapies. Tissue inhibitor of matrix metalloproteinases 4 (TIMP4) is associated with cardiovascular functioning and disease and has been linked to vascular dementia. Using immunohistochemistry, we studied occipital brain tissue samples of 57 patients with CAA (39 without ICH and 18 with ICH) and 42 controls, and semi-quantitatively assessed expression levels of TIMP4. Patients with CAA had increased vascular expression of TIMP4 compared to controls (*p* < 0.001), and in these patients, TIMP4 expression correlated with CAA severity (τ_b_ = 0.38; *p* = 0.001). Moreover, TIMP4 expression was higher in CAA-ICH compared to CAA-non-ICH cases (*p* = 0.024). In a prospective cross-sectional study of 38 patients with CAA and 37 age- and sex-matched controls, we measured TIMP4 levels in cerebrospinal fluid (CSF) and serum using ELISA. Mean CSF levels of TIMP4 were decreased in patients with CAA compared to controls (3.36 ± 0.20 vs. 3.96 ± 0.22 ng/ml, *p* = 0.033), whereas median serum levels were increased in patients with CAA (4.51 ng/ml [IQR 3.75–5.29] vs 3.60 ng/ml [IQR 3.11–4.85], *p*-9.013). Moreover, mean CSF TIMP4 levels were lower in CAA patients who had experienced a symptomatic hemorrhage compared to CAA patients who did not (2.13 ± 0.24 vs. 3.57 ± 0.24 ng/ml, *p* = 0.007). CSF TIMP4 levels were associated with CSF levels of Aβ40 (spearman r (r_s_) = 0.321, *p* = 0.009). In summary, we show that TIMP4 is highly associated with CAA and CAA-related ICH, which is reflected by higher levels in the cerebral vasculature and lower levels in CSF. With these findings we provide novel insights into the pathophysiology of CAA, and more specifically in CAA-associated ICH.

**Supplementary Information:**

The online version contains supplementary material available at 10.1186/s40478-024-01823-x.

## Introduction

Cerebral amyloid angiopathy (CAA) is a cerebral small vessel disease characterized by the progressive accumulation of the amyloid β (Aβ) protein in cerebral vessel walls, and is associated with an increased risk of developing cognitive impairment and intracerebral hemorrhages (ICH) [20]. CAA is very prevalent; 23% of people over the age of 55 will develop moderate-to-severe CAA [13], and, to date, no treatment is available to cure CAA or stop its progression. CAA is clinically diagnosed using the Boston Criteria 2.0, which relies on the presence of hemorrhagic (strictly lobar microbleeds and cortical superficial siderosis) and non-hemorrhagic (severe perivascular spaces in centrum semiovale or white matter hyperintensities in multisport pattern) markers on MRI [3]. However, these criteria detect only late-stage manifestations of CAA, and can only detect CAA with ‘probable’ or ‘possible’ likelihoods. More insight into the molecular mechanisms associated with CAA pathology is urgently needed, in order to obtain novel leads for the development of targeted therapies.

Matrix metalloproteinases (MMPs) are a family of endopeptidases with more than 20 identified members, that are capable of degrading different extracellular matrix proteins such as collagen and gelatin. Tissue inhibitors of metalloproteinases (TIMPs) regulate the activity of MMPs. Four TIMPs exist, sharing 40% sequence homology with each other [25]. In contrast to TIMP1, TIMP2, and TIMP3, which are ubiquitously expressed, TIMP4 is predominantly expressed in the brain and heart [21, 23, 25]. Evidence suggests that TIMP4 is highly associated with cardiovascular functioning and disease, and that TIMP4 plays a role in inflammatory processes of human cardiovascular pathology [15].

Increased levels of TIMP4 have been measured in plasma of Alzheimer’s dementia patients [24, 30], even in early stages of disease [14]. Increased plasma levels of TIMP4 have also been linked to vascular dementia [18]. We have previously shown that levels of MMP9 and TIMP3 are increased in the vasculature of CAA patients. Moreover, we have demonstrated aberrant levels of MMP9 and TIMP3 in the vasculature of CAA patients that suffered from ICH compared to CAA patients without ICH. The association between TIMP4 and CAA has not been previously investigated, although the cardiovascular functional specificity of TIMP4 suggests that this protein may play a role in the pathophysiology of CAA. Therefore, we set out to investigate the potential association of TIMP4 with CAA, and the relation with CAA-related ICH. We performed immunohistochemistry to assess TIMP4 expression in human brain tissue of CAA patients (with and without haemorrhage) and compared this to control cases. Furthermore, to evaluate the potential diagnostic use during life, using ELISA we measured blood and cerebrospinal fluid (CSF) TIMP4 levels in patients with CAA (with and without haemorrhage) and control participants.

## Methods

### Human brain tissue cohort

Human post-mortem formalin-fixed and paraffin-embedded (FFPE) occipital lobe tissue was obtained from Radboudumc Nijmegen, the Netherlands Brain bank (NBB; Amsterdam), the University Medical center Utrecht (UMCU), and the NeuroBiobank of the Institute Born-Bunge (NBB-IBB; Wilrijk (Antwerp), Belgium). In total, 57 CAA cases (39 non-hemorrhagic cases [CAA-NH] and 18 cases with ICH in lobar locations [CAA-ICH]) were selected based on the presence of moderate to severe CAA according to neuropathological assessments in autopsy reports. Tissue of 42 age- and sex-matched control cases without neurological disorders or amyloid pathology as per autopsy reports was also included (Table 1). We investigated occipital lobe tissue, as this brain region is generally most severely affected by CAA [2].

**Table 1:** Human brain tissue samples

	Control	CAA-NH	CAA-ICH	*p* value
n	42	39	18^*^	
Age (mean ± sd)	78.4 (± 8.4)	77.5 (± 11.9)	78.3 (± 7.6)	0.90^a^
Sex (% female)	52.4	51.3	61.1	0.77^b^
CAA grade (mean ± sd)	0.7 (1.1)	3.3 (0.9)	3.6 (0.6)	0.52^c^
Brain bank origin	16.7% NBB-IBB 40.5% NBB 42.9% RUMC	15.4% NBB-IBB 41.0% NBB 43.6% RUMC	33.3% NBB-IBB 33.3% NBB 27.8% RUMC 5.6% UMCU	0.58^b,d^
AD pathology reported (%)^#^	N.A	74%	67%	0.55^b^
Large vessel atherosclerosis reported (%)^$^	N.A	41%^f^	41%^e^	0.97^b^

CAA-NH, CAA non-haemorrhagic; CAA-ICH, CAA-associated intracerebral haemorrhage; sd, standard deviation; N.A., not assessed; RUMC, Radboud university medical center; NBB-IBB, NeuroBioBank of the Institute Born-Bunge; NBB, Netherlands Brain bank; UMCU, University Medical Center Utrecht. Tested with ^a^One-Way ANOVA, ^b^Pearson Chi-Square, ^c^Mann Whitney. ^d^UMCU and Radboudumc cases were pooled for statistical analyses. ^e,f^Information unavailable for ^e^one or ^f^two cases. ^#^The presence of AD pathology was defined as the presence of AD pathological hallmarks to a degree that they match a clinical diagnosis of AD according to board-certified neuropathologists judgements (generally at least Braak stage 4B). ^$^As reported in neuropathological assessments in autopsy reports. ^*^Occipital tissue was replaced by frontal tissue for 1 NBB CAA-ICH case

Brain tissue was used in accordance with the local medical research ethics committee of the UMCU and Radboudumc, reference numbers 17–092 and 2015–2215, respectively. The brain donor program of the NBB is approved by Medical Ethics Committee of the VU Medical Centre (Ref. No. 2009/148). The Neurobiobank of the Institute Born-Bunge (NBB-IBB) with FAMHP registration ID BB190113 is subject to biannual evaluation by the local Ethics Committee of University hospital Antwerp (UZA)/University of Antwerp (UAntwerp), Belgium, approval reference 19/13/166.

### Immunohistochemical analysis of human brain tissue

Since the occipital lobe is generally earliest and most severely affected by CAA [2], we stained one occipital lobe section of every case for TIMP4 and one for Aβ. Tissue sections (4 µm) were deparaffinized and incubated with 3% H_2_O_2_ in methanol (15 min at room temperature (RT)) to block endogenous peroxidase activity. Sections were washed three times with PBS after each of the following steps. For Aβ staining, antigen retrieval was achieved by 5 min incubation with neat formic acid. In addition, for all sections heat-induced antigen retrieval was performed in citrate buffer (pH 6.0). Sections were incubated with 5% normal horse serum (for TIMP4) or rabbit serum (for Aβ) in PBS (30 min at RT). Sections were incubated overnight at 4°C with mouse-anti-TIMP4 (Sigma-Aldrich, MABC1142, diluted 1:800 in PBS, the antibody shows no cross reactivity with TIMP1, TIMP2, TIMP3 [8]) or mouse-anti-Aβ (Biolegend, 800701, diluted 1:4000 in PBS). This was followed by incubation with biotinylated horse-anti-mouse for TIMP4 (Vector, BA2000, diluted 1:200 in PBS) or rabbit-anti-mouse for Aβ (Abcam, Ab97044, diluted 1:500 in PBS), 30 min at RT. Then, sections were incubated 30 min at RT with an avidin/biotin horseradish peroxidase complex (Vector; cat. no. PK-6100). Finally, sections were incubated 7 min with 3,3’-diaminobenzidine substrate, prepared according to the manufacturer’s protocol (Vector, cat. no. PK4100). After staining with haematoxylin, sections were dehydrated and mounted with Quick D or Pertex mounting medium. Appropriate negative controls were included by omitting the primary antibody. Sections were stained during multiple runs. Therefore, internal controls were included in every batch to ensure there was minimal inter-run variation.

Vascular TIMP4 scores were provided by two independent raters who were blinded to diagnosis of CAA and presence of prior ICH. In case of disagreement a third rater was consulted. Each section was assessed in a semiquantitative manner using a four-point scale (score 0–3). A score of 0 indicated no TIMP4-positive vessels, a score of 1 indicated mild TIMP4 staining (0–25% of vessels stained to some degree), a score of 2 indicated moderate TIMP4 staining (25–75% of vessels stained to some degree), and a score of 3 indicated severe TIMP4 staining (> 75% of vessels stained to some degree; supplementary Fig. 1). CAA burden in the studied sections was determined (according to Olichney et al. [22]) by two independent raters blinded to diagnosis of CAA and presence of prior ICH, who consulted a third rater in case of disagreement (supplementary Fig. 2). The presence of large atherosclerosis was documented when reported in autopsy reports. The presence of Alzheimer’s disease (AD) pathology was defined as the presence of AD pathological hallmarks to a degree that they match a clinical diagnosis of AD according to board-certified neuropathologists judgements (generally at least Braak stage 4 with CERAD stage B). To study the potential relationship of TIMP4 with AD pathology in more detail, TIMP4-stained tissue sections of 21 AD patients were compared with (semi)-adjacent sections stained for Aβ to assess whether TIMP4 could be observed in Aβ plaques.

### Participant cohort for body fluid biomarker analysis

We included 38 patients with probable CAA according to the modified Boston criteria [19] from Radboud University Medical Center (RUMC) (Nijmegen). Cerebrospinal fluid (CSF) and serum was collected in the context of a cross-sectional study investigating new CSF biomarkers for CAA (BIONIC; Biomarkers for Cognitive Impairment Due to Cerebral Amyloid Angiopathy; http://www.radboudumc.nl/BCS [6, 28]). Patients were consecutively recruited from the neurology and geriatric outpatient clinic from the RUMC between December 2018 and December 2022. Exclusion criteria were contra-indications for lumbar puncture or MRI, and recent (< 3 months) symptomatic stroke. Five included patients had experienced a symptomatic ICH, which had occurred 5, 16, 17, 92, and 192 months prior to lumbar puncture.

We included 37 age- and sex-matched control participants. Of 28 participants, CSF and serum was collected in the context of the CAFE (Cerebral Amyloid angiopathy Fluid biomarkers Evaluation; http://www.radboudumc.nl/BCS) study. They were recruited at the RUMC from partners and family of the patients with sCAA, or via the Dutch Brain Research Registry [32]. Inclusion criteria were age ≥ 55 years, a Montreal Cognitive Assessment (MoCA) score of > 28 or a modified Telephone Interview of Cognitive Status (mTICS) score of ≥ 35 [5, 26]. Additional exclusion criteria for the controls included self-reported (subjective) cognitive decline, and a history of major brain pathology such as spontaneous parenchymal intracerebral hemorrhage, ischemic stroke, neurodegenerative disease, brain tumors, brain infection or inflammation. In addition, CSF was collected from nine other control subjects, who underwent a lumbar puncture as part of diagnostic workup to exclude central nervous system involvement of a systemic disease, a (central) neurological cause for their symptoms, or a neurological infection or inflammation. Exclusion criteria were neurodegenerative disease, known cognitive impairment, sepsis, a recent stroke (< 6 months), and a malignancy in the central nervous system.

### Biomarker quantification in cerebrospinal fluid and serum

CSF was obtained via lumbar puncture and collected in polypropylene tubes, centrifuged, aliquoted, and stored in polypropylene tubes at − 80 °C. Serum was obtained via venipuncture and collected in polypropylene tubes, centrifuged, aliquoted, and stored in polypropylene tubes at − 80°C. TIMP4 levels were determined in CSF and serum using an ELISA kit (R&D DuoSet, R&D Systems, Minneapolis, MN, USA), according to the manufacturer’s instructions. CSF samples were 12 × diluted and serum samples were 16 × diluted; samples were added to the plate at a final volume of 100 µl and measured in duplicate. Serum and CSF albumin levels were determined using nephelometric assays using an Atellica Neph 630 analyzer. CSF levels of Aβ40, Aβ42, tau phosphorylated at threonine 181 (p-tau), and total tau (t-tau) were quantified using Lumipulse chemiluminescent immunoassays (Fujirebio, Ghent, Belgium), as previously described [6]. We stratified our cohort based on the presence or absence of a CSF biomarker profile indicative of concomitant AD according to the ATN classification system (i.e. amyloid (A)/tau (T) /neurodegeneration (N) [12]). An ATN + profile indicative of AD pathology was defined as a decrease of Aβ42 and an increase of phosphorylated tau181 and total tau, using locally defined cut-off values for Aβ42 (< 659 pg/mL), phosphorylated tau181 (> 64 pg/mL), and total tau (> 400 pg/mL). For a subset of participants (14 controls and 11 CAA patients; supplementary Table 1), CSF MMP2, MMP9, and MMP14 levels had been previously determined [28].

### MRI acquisition and quantification of cerebral microbleeds (CMBs) and cortical superficial siderosis (cSS)

As part of the BIONIC and CAFE studies, participants underwent brain MRI on a 3.0 Tesla MRI scanner (Siemens Magnetom Prisma, Siemens Healthineers, Erlangen, Germany) using a 32-channel head coil. The MRI protocol included a 3D multi-echo gradient echo T2*-weighted sequence, amongst other sequences. Magnitude and phase data from the multi-echo gradient sequence was processed to a SWI using the “Contrast-weighted, Laplace-unwrapped, bipolar multi-Echo, ASPIRE-combined, homogeneous, improved Resolution SWI” (CLEAR-SWI) method [9]. The number of lobar cerebral microbleeds (CMBs) [10] and presence of cortical superficial siderosis (cSS) [19] were rated by two trained raters in case of disagreement, a senior vascular neurologist was consulted to reach final consensus. Patients were categorized based on the observed number of lobar microbleeds (0–1 CMBs = cat 0; 2–4 CMBs = cat 1; 5–9 CMBs = cat 2; 10–19 CMBs = cat 3; 20–99 CMBs = cat 4; ≥ 100 CMBs = cat 5). cSS was categorized as either present or absent.

### Statistical analysis

Data analysis was performed using IBM SPSS Statistics (Version 29.0.0.0) and visualized using IBM SPSS Statistics or Graphpad Version 9.5.0. For analysis of immunohistochemical data, a Quade’s rank analysis of covariance was performed to determine whether the TIMP4 vascular scores differed between study groups. As differences were observed between brain banks, we included tissue source into the model as confounding factor, in addition to age and sex. Since only 1 patient from UMCU was included, data regarding this tissue sample was pooled with Radboudumc tissue samples. Sensitivity analyses were performed by pooling the UMCU sample with NBB samples or NBB-IBB samples, which did not alter the outcome. Associations between TIMP4 and CAA grade were assessed using Kendall’s tau-b (τ_b_). The effect of the presence of AD pathology and atherosclerosis on TIMP4 scores in CAA cases was assessed using Quade’s rank analysis of covariance with biobank, age, and sex as covariates.

The CSF:serum ratios of albumin (Q-albumin; a measure of blood-CSF barrier integrity) and of TIMP4 (Q-TIMP4) were determined, as well as the TIMP4 index (Q-TIMP4/Q-albumin). Normality of data was assessed using Shapiro–Wilk tests. CSF levels and CSF:serum ratios were normally distributed and expressed as mean ± standard deviation (sd) and statistical analysis was performed on raw data. Serum levels of TIMP4 were heavily right-skewed, therefore, raw data is presented as median with interquartile range (IQR), while statistical analysis was performed on inverse-transformed data. TIMP4 index data was right-skewed and therefore log-transformed. To assess group differences, standard multiple regression analyses with age and sex as covariates were performed.

To assess correlations of CSF or serum TIMP4 with CSF Aβ40, CSF Aβ42, or MoCA score, partial Spearman rank correlations (r_s_) were applied with age and sex as covariates. Linear regression analysis with age and sex as covariates was performed to assess the effect of hypertension on CSF TIMP4. In CAA patients, potential differences in CSF TIMP4 between patients without and with symptomatic ICH were assessed with Mann–Whitney U test, the association between lobar microbleeds category and CSF TIMP4 levels was assessed using partial Spearman rank correlation with age and sex as covariates, and the association between CSF TIMP4 levels and the presence of cSS was assessed with linear regression analysis with age and sex as covariates. Linear regression analysis with age and sex as covariates was performed to assess whether TIMP4 levels differed between CAA patients with or without an ATN + CSF biomarker profile and between CAA patients with or without at least one APOE ε4 allele.

Potential differences in MMP:TIMP4 ratios between controls and CAA patients were assessed with Mann–Whitney U test, since these subgroups were deemed too small for inclusion of potential confounders into statistical analyses. Areas under the curve (AUC) were determined using a receiver operating characteristic curve (ROC) with 95% confidence interval (CI) to calculate the capacity of CSF and serum TIMP4 levels to discriminate CAA patients from controls. We also assessed whether the addition of CSF TIMP4 improved the diagnostic accuracy of other CSF markers for CAA (Aβ40, Aβ42, p-tau, t-tau).

## Results

### Increased TIMP4 levels in cerebral vessels in brains with CAA-related intracerebral hemorrhage

**Fig. 1:**
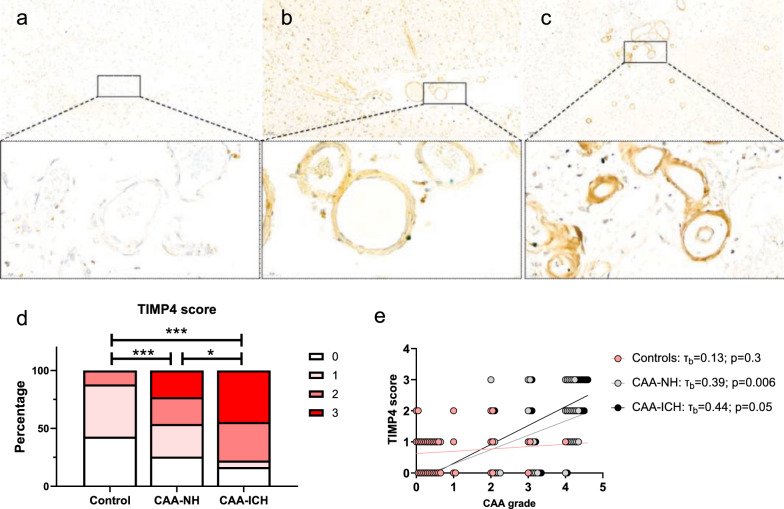
Immunohistochemical analysis of TIMP4 in human brain tissue. **a–c** Representative examples of vascular TIMP4 staining in control (**a**), CAA-NH (**b**), and CAA-ICH (**c**) tissue. Upper panels show microscopic images at 10× magnification (scalebar = 100 µm), lower panels show depicted area at 63× magnification (scalebar = 20 µm). **d** Both CAA-NH and CAA-ICH cases had significantly higher TIMP4 scores compared to controls, and in addition, CAA-ICH cases had higher TIMP4 scores than CAA-NH cases. **e** TIMP4 score correlated to CAA grade in the CAA-NH and CAA-ICH groups (**e**). *p ≤ 0.05; ***p ≤ 0.001

Compared to controls (mean 0.7 ± 0.7, Fig. 1a, d), both CAA-NH (1.4 ± 1.1; *p* < 0.001, Fig. 1b, d) and CAA-ICH (2.1 ± 1.1; *p* < 0.001, Fig. 1c,d) groups had increased TIMP4 scores. Furthermore, CAA-ICH cases had higher TIMP4 scores compared to CAA-NH cases (*p* = 0.024, Fig. 1d).

In controls, TIMP4 score did not correlate to CAA grade (τ_b_ = 0.14; *p* = 0.31, Fig. 1e), in contrast to the combined CAA group (τ_b_ = 0.38; *p* = 0.001). Also, in the separate CAA-NH (τ_b_ = 0.39; *p* = 0.006) and CAA-ICH groups (τ_b_ = 0.44; *p* = 0.05), TIMP4 score correlated with CAA grade (Fig. 1e). The presence of atherosclerosis (*p* = 0.7) did not have an effect on TIMP4 score in CAA patients. Similarly, the presence of AD pathology did not influence TIMP4 score in CAA patients (τ_b_ = *p* = 0.3). In line with that, qualitative comparison of TIMP4-stained sections with (semi)-adjacent Aβ-stained sections did not reveal the presence of TIMP4 in amyloid plaques in any of the studied AD cases (supplementary Fig. 3).

### Altered TIMP4 levels in CSF and serum in CAA

Of 4 CAA participants, no CSF samples were available due to unsuccessful lumbar puncture, and of 1 CAA participant, no CSF measurement was performed. In addition, no serum measurement was performed for 1 CAA patient. Therefore, a total of 33 CSF measurements and 37 serum measurements were included for analysis. Of 1 control participant, there was no CSF sample available due to unsuccessful lumbar puncture, of 1 control participant, no CSF measurement was performed, and of 9 control participants, no serum was collected. TIMP4 measurements in one serum sample had a high coefficient of variation (44%), but since this did not influence the statistical results, the sample was included for analysis. Therefore a total of 35 CSF measurements and 28 serum measurements from control participants were included for analysis (Table 2).

**Table 2:** Cohort for body fluid biomarker assessment

	Control	CAA	*p*
N total (CSF/serum/CSF: serum)	37 (35/28/26)	38 (33/37/32)	–
Mean age, years (sd)	71.1 (6.2)	72.0 (6.2)	0.55^a^
Sex (% female)	51%*	45%	0.65^b^
Hypertension (%)	50%*	61%	0.46^b^
MoCA score (median [IQR])	28 [27–29]*	24.5 [21–27.25]	< 0.001^c^
ApoE ε4 carrier	25%*	50%	0.046^b^
* Heterozygous*	25%	29%	–
* Homozygous*	0%	21%	–
Q-Albumin (mean (sd))	6.5 (0.3)	6.5 (0.4)	0.80^a^
CSF Aβ40, ng/ml (mean (sd))	12.28 (4.11)	8.13 (2.19)	< 0.001^a^
CSF Aβ42, ng/ml (mean (sd))	1.01 (0.40)	0.42 (0.17)	< 0.001^a^
CSF t-tau, pg/ml (mean (sd))	365 (29)	510 (52)	0.007^a^
CSF p-tau, pg/ml (mean (sd))	47 (4)	65 (8)	0.015^a^
Number of lobar CMBs (median [IQR])	0 [0–0]	8 [4–23]	< 0.001^c^
Presence of cSS (%)	0%	71%	< 0.001^b^

Characteristics for the complete control and CAA groups are shown. No CSF TIMP4 measurements were available for 2 controls and 5 CAA patients. No serum TIMP4 measurements were available for 9 controls and 1 CAA patient. No CSF: serum ratios could be determined for 11 controls and 6 CAA patients. CAA, cerebral amyloid angiopathy; CSF, cerebrospinal fluid; MoCA, Montreal Cognitive Assessment; sd, standard deviation; IQR, interquartile range; CMBs, cerebral microbleeds; cSS, cortical superficial siderosis. Assessed with ^a^t-test, ^b^Chi-squared test, and ^c^Mann-Whitney U test. *Data on hypertension, MoCA score, and ApoE genotype was only available for the 28 control participants included in CAFE

**Fig. 2:**
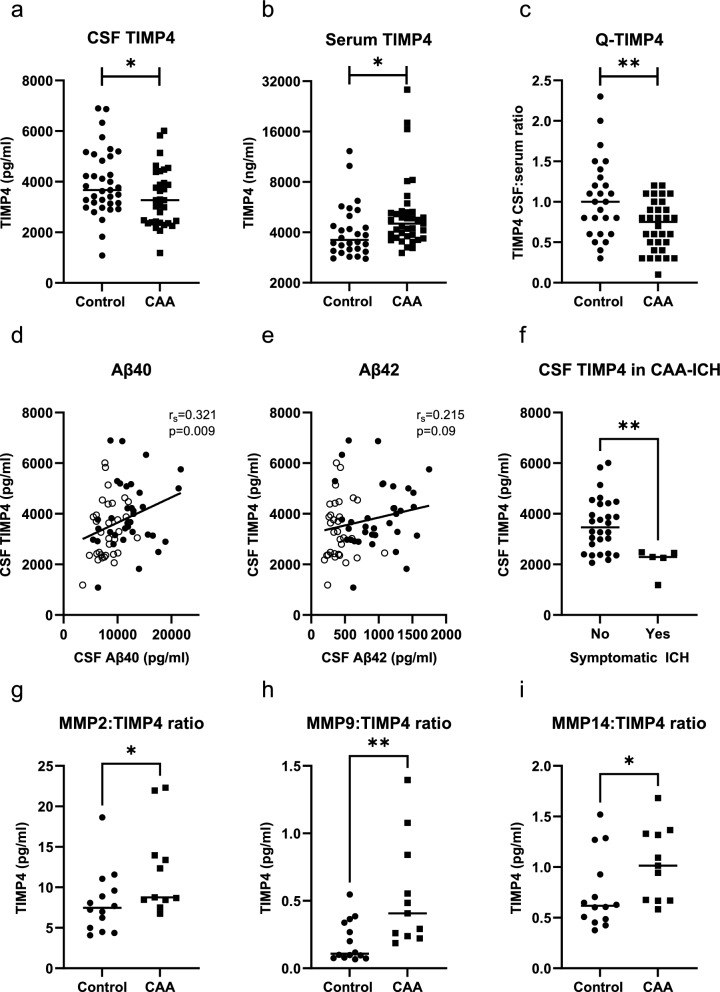
TIMP4 levels in CSF and serum.** a** CSF TIMP4 levels were decreased in patients with CAA compared to controls. **b** Serum TIMP4 levels were increased in patients with CAA compared to controls (y-axis has a log2 scale for visualization purposes). **c** The CSF:serum ratio of TIMP4 (Q-TIMP4) was lower in patients with CAA than controls. **d** CSF TIMP4 levels were associated with CSF levels of Aβ40 in the combined group of patients with CAA and controls. **e** CSF TIMP4 levels were not associated with CSF levels of Aβ42 in the combined group of patients with CAA and controls. **d**–**e**: Open circles: patients with CAA; closed circles: controls. **f** CSF TIMP4 levels were decreased in patients that had experienced a symptomatic ICH. **g**–**i** The MMP2:TIMP4, MMP9:TIMP4, and MMP14:TIMP4 ratios were increased in patients with CAA compared to controls. CAA, cerebral amyloid angiopathy; ICH, intracerebral haemorhage; CSF, cerebrospinal fluid. *p ≤ 0.05; **p ≤ 0.01

Mean CSF TIMP4 levels were lower in patients with CAA (3.36 ng/ml ± 0.20) compared to controls (3.96 ng/ml ± 0.22, β =  − 0.26, *p* = 0.033; Fig. 2a). In contrast, median serum TIMP4 levels were higher in patients with CAA (4.51 ng/ml [IQR 3.75–5.29]) compared to controls (3.60 ng/ml [IQR 3.11–4.85], inverse transformed β =  − 0.30, *p* = 0.013; Fig. 2b). The mean CSF:serum ratio of TIMP4 (Q-TIMP4) was lower in patients with CAA (0.71 ± 0.05) compared to controls (1.04 ± 0.10, β =  − 0.38, *p* = 0.003; Fig. 2c). The median TIMP4 index (Q-TIMP4/Q-albumin) was also lower in patients with CAA (0.11 [IQR 0.07–1.6]) compared to controls (0.15 [IQR 0.10–0.22], log-transformed β =  − 0.31, *p* = 0.022).

CSF TIMP4 levels were associated with CSF levels of Aβ40 (r_s_ = 0.321, *p* = 0.009, Fig. 2d), but not with Aβ42 (r_s_ = 0.215, *p* = 0.09, Fig. 2e) in the combined group of patients with CAA and controls. No significant correlations between CSF TIMP4 levels and CSF levels of Aβ40 (control: r_s_ = 0.07, *p* = 0.71; CAA: r_s_ = 0.29, *p* = 0.12) or Aβ42 (control: _rs_ = 0.03, *p* = 0.86; CAA: r_s_ = 0.14, *p* = 0.47) were observed in the separate groups of controls or CAA patients. Neither CSF TIMP4 levels (r_s_ = 0.20, *p* = 0.27) nor serum TIMP4 levels (r_s_ =  − 0.01, *p* = 0.97) were associated with MoCA score in CAA patients. Furthermore, CSF TIMP4 levels were not associated with the presence of hypertension (complete cohort: β = 0.03, *p* = 0.84; control: β =  − 0.09, *p* = 0.72; CAA: β = 1.44, *p* = 0.46).

CSF TIMP4 levels did neither differ between CAA patients with or without an ATN + CSF biomarker profile (3.6 ± 0.3 vs 3.2 ± 0.2 ng/ml; β = 0.20, *p* = 0.30) nor between CAA patients with or without at least one APOE ε4 allele (3.8 ± 0.6 vs 4.0 ± 0.3 ng/ml; β = 0.06, *p* = 0.76).

Remarkably, in the five patients with CAA that had experienced at least one symptomatic ICH, lower CSF TIMP4 levels (2.13 ng/ml ± 0.24) were observed compared to CAA patients without symptomatic hemorrhage (3.57 ng/ml ± 0.20; *p* = 0.007, Fig. 2f). In line with this, in CAA patients CSF TIMP4 levels negatively correlated to the number of lobar CMBs (r_s_ =  − 0.39, *p* = 0.031). In contrast, the presence of cSS was not associated with altered CSF TIMP4 levels (β = 0.13, *p* = 0.49).

For a subset of samples (11 CAA samples and 14 control samples), data on CSF levels of MMP2, MMP9, and MMP14 was available. This subset of CSF samples was respresentative of the larger group with regard to lower TIMP4 levels in patients compared to controls (Supplementary Table 1). The ratios of MMP2:TIMP4 (*p* = 0.038, Fig. 2g), MMP9:TIMP4 (*p* = 0.005, Fig. 2h), and MMP14:TIMP4 (*p* = 0.025, Fig. 2i) were increased in patients with CAA compared to controls.

ROC analysis (probable CAA vs controls) yielded an AUC value of 0.64 [95%CI 0.51–0.77] for CSF TIMP4. The addition of CSF TIMP4 to CSF levels of Aβ40 and Aβ42 yielded an AUC of 0.94 [0.89–0.99] compared to an AUC of 0.93 [0.87–0.99] for CSF Aβ40 and of Aβ42 alone. A combination of CSF TIMP4 with CSF Aβ40, Aβ42, p-tau, and t-tau yielded an AUC of 0.97 [0.93–1.00], compared to an AUC of 0.96 [0.91–1.0] for CSF Aβ40, Aβ42, p-tau, and t-tau alone. Serum TIMP4 had an AUC of 0.59 [0.50–0.69] and the CSF:serum ratio of TIMP yielded an AUC of 0.69 [0.55–0.82].

## Discussion

In the present study, we investigated the association of TIMP4 with CAA by studying TIMP4 levels in the brain vasculature as well as in body fluids. We observed increased levels of TIMP4 using post-mortem immunohistochemical assessment of occipital lobe vasculature in CAA patients, compared to controls. Moreover, CAA patients who had an ICH had more widespread cerebrovascular TIMP4 compared to CAA patients without ICH. These immunohistochemical findings were reflected in our observations on CSF levels of TIMP4: we found decreased CSF TIMP4 levels in CAA patients compared to controls. Moreover, CAA patients who had suffered a symptomatic ICH had significantly lower CSF levels of TIMP4 compared to CAA patients who had not suffered a symptomatic ICH.

To our knowledge, the association of TIMP4 with CAA is a novel finding. We demonstrate decreased CSF levels of TIMP4 in CAA patients compared to controls, whereas cerebrovascular levels of TIMP4 detected by immunohistochemistry are increased in CAA patients. This expression pattern is similar to that of Aβ40 (and other Aβ isoforms); increased vascular deposition of Aβ in CAA is reflected by decreased CSF levels [4, 6, 27]. Indeed, we observed a correlation between CSF levels of TIMP4 and CSF levels of Aβ40. The reduction of Aβ levels (especially of Aβ40) in CSF is thought to be the result of reduced clearance of brain Aβ towards the CSF. TIMP4 is a soluble protein and secreted extracellularly [25], and has been shown to accumulate in the vasculature after localized injury [7, 15]. Likely, CAA causes a similar pathological response, resulting in increased vascular accumulation of TIMP4 and a reduced net efflux towards the CSF.

Increased levels of TIMP4 in blood have been associated previously with both cerebral small vessel disease (cSVD) and cognitive impairment. In 225 patients with ischaemic stroke, signs of cSVD (i.e. white matter changes, lacunes, and brain atrophy) were associated with higher circulating TIMP4 levels [1]. In a cohort of 494 participants with variable burden of cSVD, higher blood levels of TIMP4 correlated to more severe white matter hyperintensities [16], a MRI marker of cSVD [29]. This suggests that TIMP4 may be involved in the pathological processes of various forms of cerebral small vessel disease, with either amyloidogenic or ischemic cause. TIMP4 has been shown to accumulate locally within arterial walls during inflammatory processes as a response to atherosclerosis and giant cell arteritis [15]. In rats, vascular TIMP4 protein levels and localized TIMP4 gene expression increased in response to carotid balloon injury (that damaged the endothelium and stretched the arterial wall media) [7]. It is thought that TIMP4 may have a protective role in response to vessel damage by inhibiting extracellular matrix (ECM) degradation and by reducing smooth muscle cell migration [7]. Our immunohistochemical data shows that a higher grade of CAA correlates to increased cerebrovascular TIMP4 levels, suggesting that TIMP4 expression may also be upregulated in response to Aβ accumulation in CAA.

Several studies have demonstrated increased plasma TIMP4 levels in AD patients compared to controls [24, 30]. Increased plasma TIMP4 levels have been measured (at baseline) in patients with mild cognitive impairment (MCI) that converted to dementia within 5 years, in comparison to MCI patients that did not progress [14]. Moreover, higher baseline plasma TIMP4 levels in dementia-free people are associated with long-term risk of dementia [18]. We show that TIMP4 levels in the brain correlate to CAA grade but not to the presence of parenchymal AD pathology. Since 80% of AD patients also have CAA [13], and TIMP4 has been particularly associated with cardiovascular processes [7, 11, 15], one may speculate that TIMP4 is linked to Alzheimer’s dementia through disturbed vascular integrity associated with CAA [14]. In line with this hypothesis, we observed increased serum levels of TIMP4 in CAA patients. Interestingly, one study previously showed that increased plasma TIMP4 levels are associated with vascular dementia but not with non-vascular dementia [18]. On the other hand, another study demonstrated lower levels of plasma TIMP4 in patients with AD (probably many with CAA) versus patients with vascular dementia [24], leaving the exact relationship between vascular damage in AD and altered TIMP4 levels somewhat unclear.

Interestingly, our immunohistochemical data demonstrated higher vascular levels of TIMP4 in patients with CAA-related ICH compared to CAA-non haemorrhagic patients. Since higher vascular TIMP4 scores were associated with higher CAA grades, it is likely that TIMP4 is retained in the vasculature due to the presence of more Aβ. Cerebrovascular Aβ has been shown to induce expression and activation of matrix metalloproteinases, including MMP9 [17, 31]. We speculate that the increased levels of MMPs stimulate the presence of TIMP4. Moreover, very low CSF TIMP4 levels were measured in CAA patients that had experienced a symptomatic haemorrhage in the past. This may indicate that in these patients, CSF TIMP4 levels are decreased due to increased retention of TIMP4 in the vasculature as a response to processes such as local inflammation. Notably, ICH had occurred 5–192 months prior to CSF withdrawal, indicating chronically reduced CSF TIMP4 levels in CAA-ICH patients rather than an acute response.

For a subset of patients, CSF levels of MMP2, MMP9, and MMP14, as well as of TIMP1, TIMP2, and TIMP3 had been determined previously [28]. Interestingly, TIMP4 was the only TIMP with decreased CSF levels in CAA patients (supplementary Table 1). Levels of the other TIMPs were (significantly or trending) increased in CSF of CAA, indicating a functional difference of TIMP4 compared to the other TIMPs. We also determined the ratio of MMP2, MMP9, and MMP14 to TIMP4 in this subset of samples. Ratios were consistently increased in CAA patients compared to controls, which is also strikingly different from observations our group made earlier regarding decreased ratios of MMPs to TIMP1, TIMP2, and TIMP3 in CSF of CAA patients. The increased MMP:TIMP4 ratios in CAA indicate an altered balance, with relatively more MMP levels compared to TIMP4 levels. This may indicate relatively higher proteolytic activity of MMPs, resulting in ECM breakdown and vulnerable vasculature.

Strengths of our study include the unique patient populations including both the large sample size for the immunohistochemical analysis as well as the large and well-characterized cohort for CSF analysis. Limitations include the heterogeneity of CAA patients included for immunohistochemical analysis, including both patients with and without concomitant AD pathology or atherosclerosis. Another limitation of the immunohistochemical analysis study is that tissue of different brain banks was included (potentially introducing differences in post-mortem interval and tissue treatment protocol) which may have affected TIMP4 staining results. However, we added brain bank as covariate into statistical analysis to account for such effects.

In summary, we show that TIMP4 is associated with CAA, which is reflected by higher cerebrovascular levels and lower CSF levels.

### Supplementary Information

Supplementary Material 1.
