# Supplementary material for: Altered brain expression and cerebrospinal fluid levels of TIMP4 in cerebral amyloid angiopathy
Source: Acta Neuropathol Commun. 2024 Jun 24;12:103. doi: 10.1186/s40478-024-01823-x (PMC11194996; doi:10.1186/s40478-024-01823-x)
Supplement: Supplementary file 1 — Supplementary Material 1. [file 40478_2024_1823_MOESM1_ESM.docx]

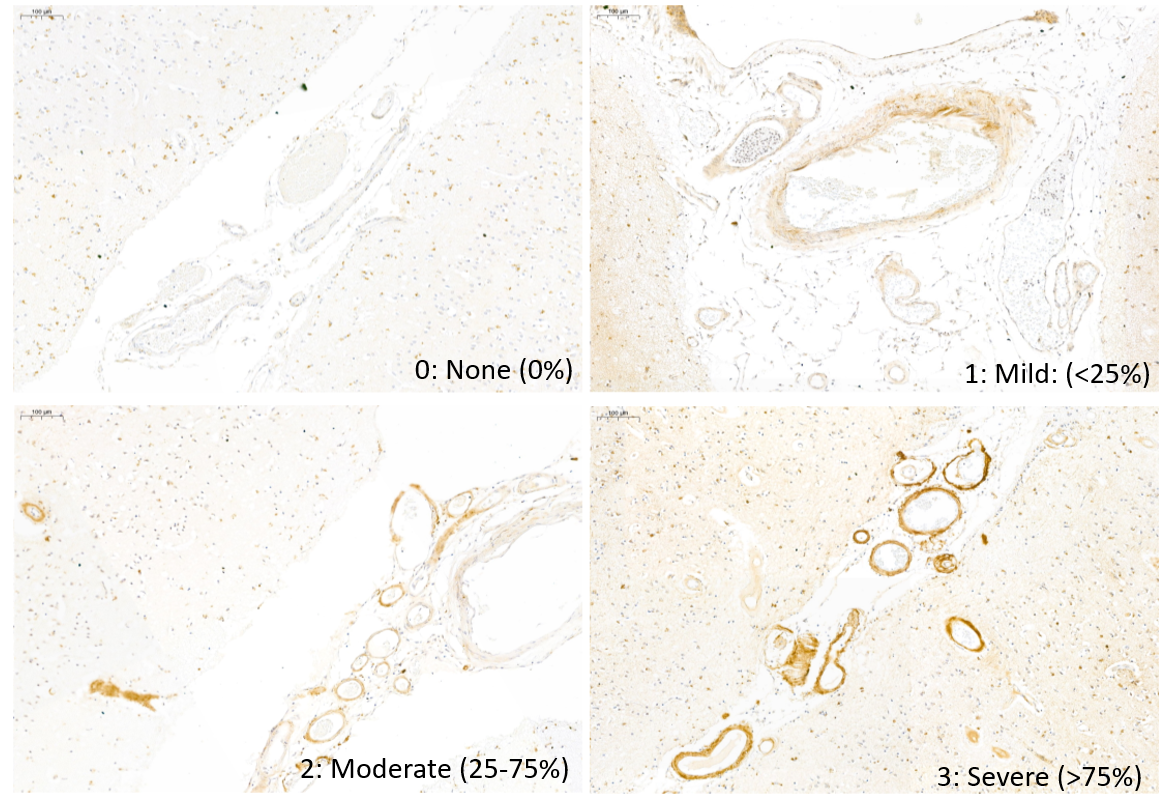


**Supplementary figure 1**: Representative examples of tissue sections with score 0 (no TIMP4-positive vessels), score 1 (mild TIMP4 staining: 0-25% of vessels stained to some degree), score 2 (moderate TIMP4 staining: 25-75% of vessels stained to some degree), and score 3 (severe TIMP4 staining: >75% of vessels stained to some degree).


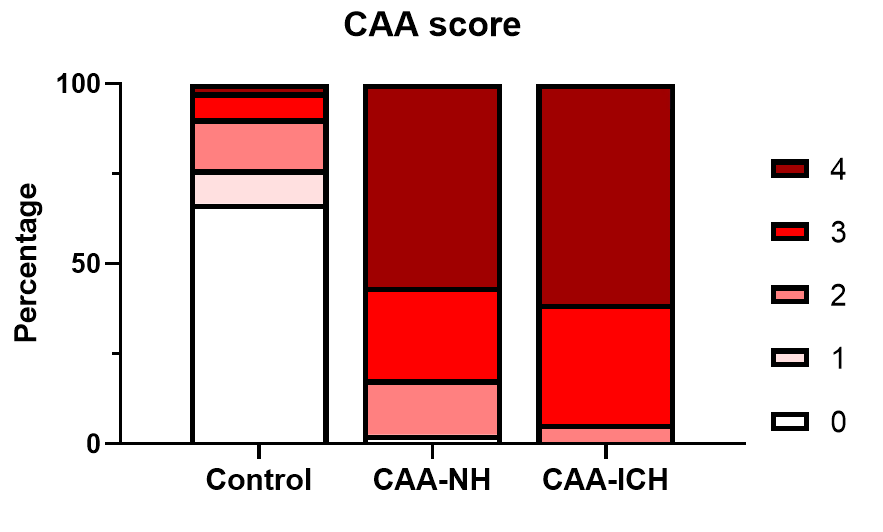


**Supplementary figure 2**: Chart displaying the distribution of CAA scores in the occipital lobe tissue of the studied groups.

**
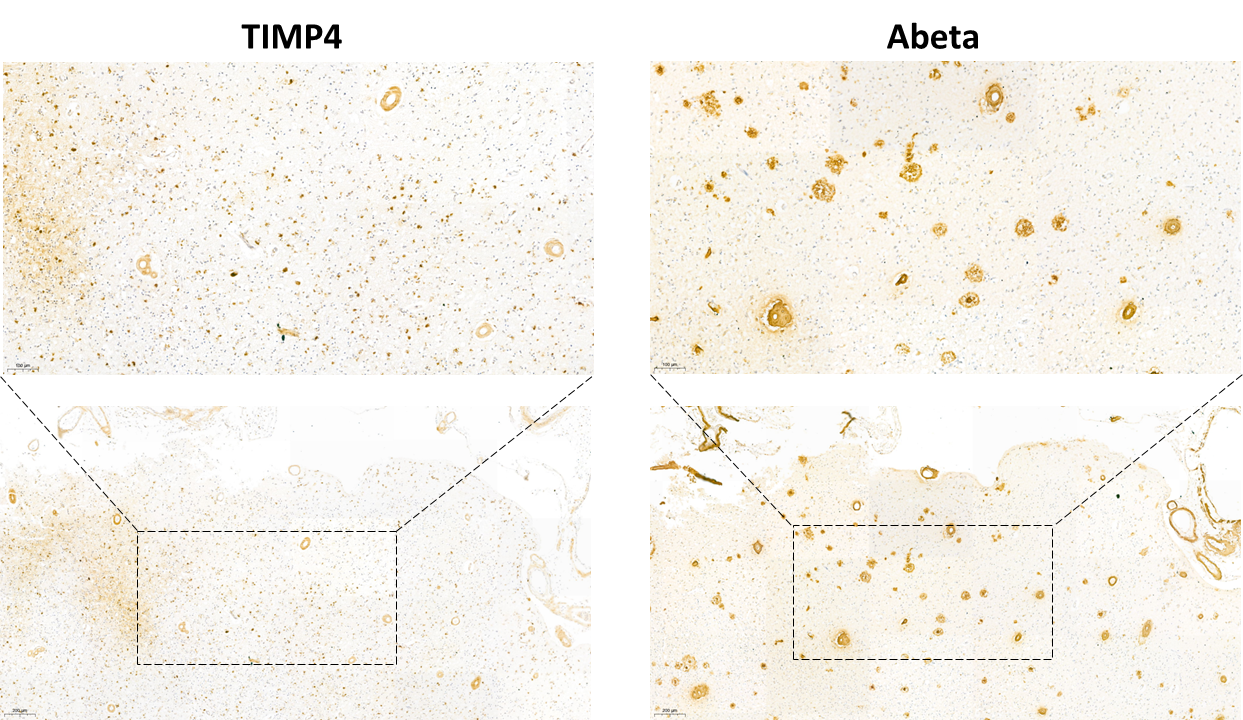
**

**Supplementary figure 3**: Adjacent sections of a representative AD case stained for TIMP4 and Aβ. TIMP4 was visible in Aβ-positive vessels, but not in Aβ-positive plaques. Scalebar upper panels = 100 µm; Scalebar lower panels = 200 µm.

**Supplementary table 1.**

|  | Control | CAA | *p* |
| --- | --- | --- | --- |
| n | 14 | 11 |  |
| Mean age (sd), years | 69.8 (6.7) | 73.8 (6.9) | 0.17^1^ |
| Sex (% female) | 71% | 73% | 1.00^3^ |
| TIMP1 (ng/mL) | 33.2 (13.9) | 45.6 (16.5) | 0.05^1^ |
| TIMP2 (ng/mL) | 45.4 (6.7) | 58.2 (14.2) | 0.007^1^ |
| TIMP3 (pg/mL) | 103.2 (44.3) | 131.8 (59.0) | 0.18^1^ |
| TIMP4 (pg/mL) | 4243 (1724) | 2897 (891) | 0.028^1^ |
| MMP2 (ng/mL) | 30.2 (10.5) | 30.9 (6.3) | 0.34^2^ |
| MMP9 (pg/ml) | 698.6 (453.8) | 1442 (990.3) | 0.009^2^ |
| MMP14 (ng/ml) | 2783 (1027) | 2741 (665) | 0.91^1^ |

For a subset of CAA patients, cerebrospinal fluid (CSF) levels of several TIMPs and MMPs had been determined previously [1]. Demographics and mean (±sd) TIMP/MMP levels are shown for this subcohort. Potential differences between controls and CAA patients were assessed with ^1^t-tests or ^2^Mann-Whitney U tests, depending on normality of data (as assessed by Shapiro-Wilk tests), or ^3^Fisher’s exact test. CAA = cerebral amyloid angiopathy; sd = standard deviation; TIMP = tissue inhibitor of matrix metalloproteinases; MMP = matrix metalloproteinase.

1 Vervuurt M, de Kort AM, Jäkel L, Kersten I, Abdo WF, Schreuder F, Rasing I, Terwindt GM, Wermer MJH, Greenberg SMet al (2023) Decreased ratios of matrix metalloproteinases to tissue-type inhibitors in cerebrospinal fluid in sporadic and hereditary cerebral amyloid angiopathy. Alzheimers Res Ther 15: 26 Doi 10.1186/s13195-023-01171-3
